# The predictive value of childhood recurrent abdominal pain for adult emotional disorders, and the influence of negative cognitive style. Findings from a cohort study

**DOI:** 10.1371/journal.pone.0185643

**Published:** 2017-09-28

**Authors:** Kate Stein, Rebecca. M. Pearson, Alan Stein, Mina Fazel

**Affiliations:** 1 Department of Psychiatry, University of Oxford, Oxford, United Kingdom; 2 School of Social and Community Medicine, University of Bristol, Bristol, United Kingdom; Southeast University Zhongda Hospital, CHINA

## Abstract

**Background:**

Recurrent abdominal pain (RAP) in childhood is common, with no explanatory pathology identified in the majority of cases. Previous studies have consistently demonstrated an association between childhood RAP and later emotional distress disorders. The aim of this study was to replicate this finding through the analysis of a large dataset, and explore how a negative style of thinking could potentially influence this relationship.

**Methods:**

The Avon Longitudinal Study of Parents and Children (ALSPAC) is a population cohort of children born in the Avon area of the UK, between 1991–1992. Data on childhood RAP was collected via maternal reports at 3, 4, 7 and 9 years. Mood, anxiety and cognitive style were measured at age 18. We controlled for various confounding factors, including maternal anxiety and the child’s pre-existing psychopathology. Logistic regression models were used to examine associations, and moderation effects of cognitive style were analysed using likelihood ratios.

**Results:**

Experiencing RAP at any one time-point is associated with an increased odds of depression and/or anxiety disorder at 18 (OR = 1.41, 95% CI 1.09–1.83). We found a dose-response relationship and each additional marker of RAP was associated with a 26% (CI: 7% to 47%) increase in risk of having a mood and/or anxiety disorder. Individuals who attribute adversity to global, stable or personal factors were at amplified risk.

**Conclusions:**

Childhood RAP predicts depression and anxiety disorders at 18 and should be targeted for early intervention. Individuals with a negative cognitive style may be particularly vulnerable, suggesting that cognitive interpretations of physical symptoms could play an important role in long-term health outcomes.

## Introduction

Recurrent abdominal pain (RAP) is one of the most common medical complaints of childhood, with no disease pathology identified in the majority of cases.[1] It affects approximately 10% of children at any one time[2, 3] and it is a frequent cause of primary care and hospital attendance.[4] The most widely accepted definition of recurrent abdominal pain, coined by Apley, is when a child has ‘had at least 3 bouts of pain, severe enough to affect his/her activities, over a period of at least 3 months, with attacks continuing in the year preceding examination.’[2] Previous studies have highlighted with relative consistency that there is an association between childhood somatic complaints and subsequent mental health problems.[5, 6] The present study aimed to first demonstrate, using a large dataset, the specific role of childhood RAP as a risk marker for anxiety and depression in early adulthood. The study also aimed to answer a novel research question: To explore the influence of a negative cognitive style on this relationship.

The importance of cognitive factors in internalising disorders, such as depression and anxiety, is well established and there is now growing interest in how cognitive factors are associated with paediatric chronic pain. Research has specifically highlighted the important role that cognitive factors play in the interpretation of bodily symptoms [7] [8] and in the experience of pain. [9] Pain catastrophizing, for example, has been shown to explain pain related disability in children with recurrent abdominal pain. [10] Whether or not cognitions need to be pain-specific to impact the pain experience is currently unknown [11] but it is known that even a general cognitive style can influence the relationship between psychological childhood stress and later depression/ anxiety. [12]

A negative cognitive style is a person’s tendency to attribute adversity to global, stable or personal factors and this may impact on their experience of pain. [13] For example, does a child believe that their abdominal pain causes problems in all areas of their life (global), or do they attribute their abdominal pain to the fact that they are ‘always more sensitive to illnesses than others’ (personal)) and do they believe that ‘they are always going to be sick’ (stable). We analysed data from the Avon Longitudinal Study of Parents and Children (ALSPAC), a large UK population-based birth cohort study of over 14,500 children. We aimed to first demonstrate the relationship between childhood RAP and later emotional distress disorders, and second examine the influence of negative cognitive style.

## Methods

### Sample

**Fig 1:**
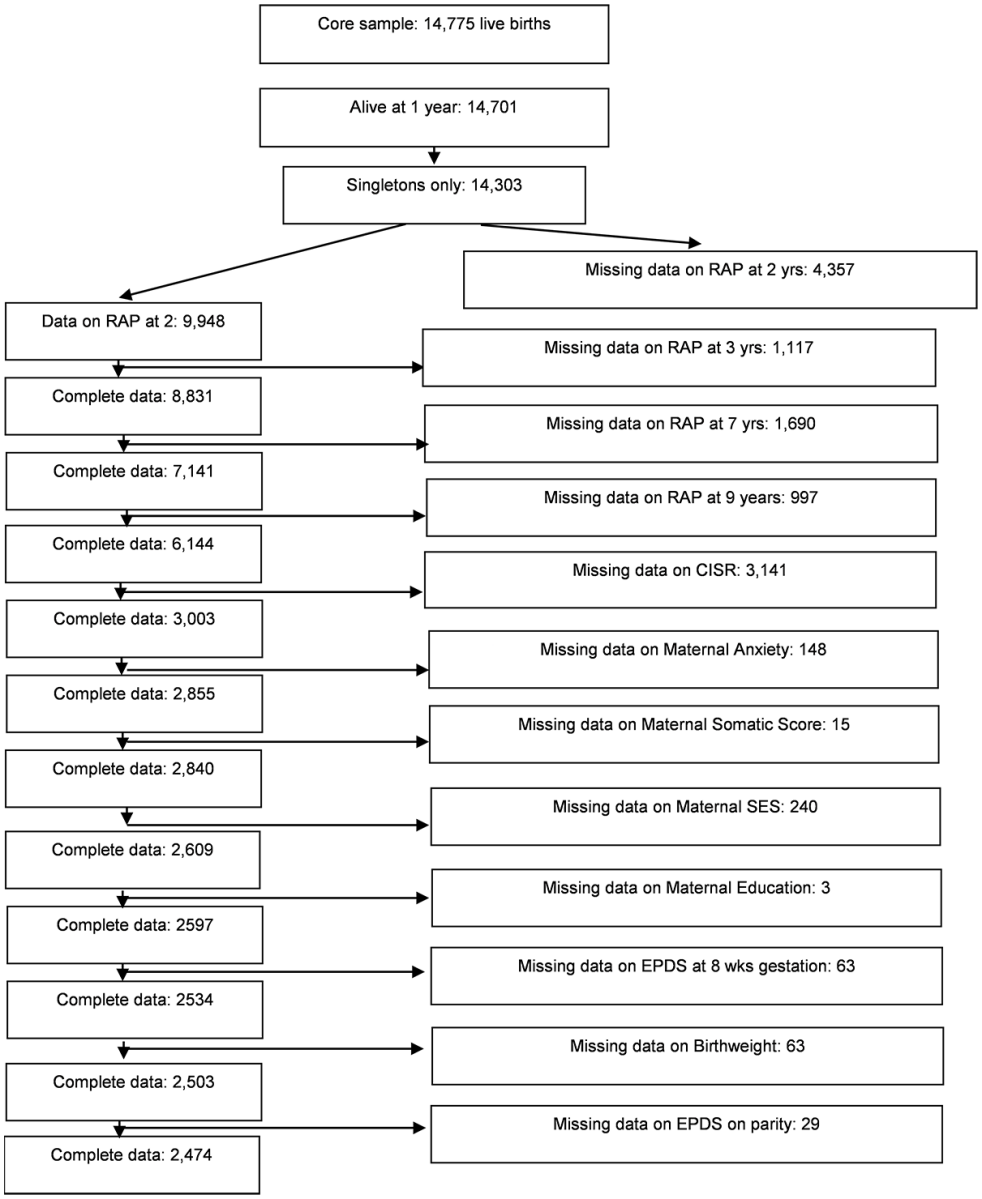
Flowchart of attrition.

The Avon Longitudinal Study of Parents and Children (ALSPAC), is a large, longitudinal, cohort study of children based in and around Bristol in the United Kingdom, the same geographic area studied by Apley and Naish(2) in their original study. The background and detailed methods of the study have been described previously (15). The children in the sample were born between 1st April 1991 and 31st December 1992, and approximately 85% of eligible women took part. The ALSPAC data set has an original sample of 14,775 live births. Children and their parents were followed up regularly through questionnaires and attendance at research clinics (see Fig 1 flowchart of attrition).

Further information is available on the study website (www.bristol.ac.uk/alspac), which includes details of all available data through a searchable data dictionary (www.bris.ac.uk/alspac/researchers/data-access/data-dictionary). Ethical approval for this study was obtained from the ALSPAC Law and Ethics Committee. Written informed consent was obtained from participants after the procedures had been fully explained.[14]

### Exposure measure

ALSPAC sent questionnaires to mothers when their children were 30 months (2 years 6 months), 42 months (3 years 6 months) and 81 months (6 years 9 months) and 103 months (8 years 7 months). Accounting for the time taken for mothers’ to report back and to add clarity and consistency, these ages have been rounded up to 3 years, 4 years, 7 years and 9 years.

ALSPAC collected childhood RAP data by asking the following questions:

1. Have there been times when he/she seems to have had a pain in his stomach in the past 12 months? (A yes/no response was required)
2. How many separate times has this happened in the past 12 months? (Responses were once, twice, 3 or 4 times, 5 times, or don’t know)

In line with previous research,[15] we included all children reported as having abdominal pain 5 times in the past year in the RAP group, because this was the group reported as having the most frequent abdominal pain and matched most closely the definition of 3 episodes of abdominal pain in 3 months used by Apley and Naish.[2] Thus, ALSPAC dataset collected data on persistence of RAP and we have used this as a proxy for severity. There were no questions about the impact of the abdominal pain on the children concerned, and our definition differs from that of Apley and Naish in this regard. However, we only included children experiencing the most frequent abdominal pain, so we consider the defined groups to be broadly comparable.

Although other somatic complaints (e.g. headache, limb ache) are common childhood, the focus of this paper is on abdominal pain because it is the most common, and ALSPAC collected the most detailed data on it. Thus, we decided to focus solely on RAP for both research and clinical reasons. Initially, we created a total RAP score, combining scores from all 4 different time points to create an ordinal figure. We then analysed separate models to investigate the association between each of the different RAP scores in turn (age 3, 4, 7 and 9 years) and depression and anxiety at age 18.

### Outcome measures

#### Depression and anxiety at age 18 years

Mood and anxiety symptoms were measured using the standardised computerized version of the Clinical Interview Schedule-Revised (CIS-R), which derives diagnoses of depression and anxiety disorders using ICD-10.[16] By measuring these symptoms at age 18, a difficult transition period where adolescents tend to move away from their parents, there could be a higher levels of emotional distress symptoms, but this would not influence our analysis of the effect of childhood RAP. The interview is fully standardized and equally reliable whether conducted by a clinically trained interviewer or self-administered on the computerised version.[16] Our study used a CIS-R score equal to/ above 12 as the cut off for our primary outcome, because this has high sensitivity and specificity in predicting clinically diagnosed depression.[17] Previous ALSPAC studies have also demonstrated significant co-morbidity between depression and anxiety at this cut off, and showed that either or both diagnoses can be given.[18] [19]

### Moderating variable

A short version of the Cognitive Style Questionnaire (CSQ) was administered at age 18.[13] [20] The CSQ is a measure of general cognitive style and focuses on eight negative hypothetical events relating to failures in academic, employment and interpersonal relationships, previous studies indicate that cognitive style is trait like and stable from childhood. For example, the CSQ may state: ‘It is my fault if I am getting along badly with my parents’. For each event, participants are told to imagine themselves in the situation and rate the extent to which the event was caused by: 1). Internal versus external factors (themselves or others), 2). Specific vs. global factors (impact all areas of life or just this specific situation), 3). Stable versus unstable factors (the cause will persist and lead to the same outcome in the future), and 4). How it reflects their self-worth. The young person answers on a Likert scale of agreement from 1–5. Higher scores indicate a more negative style, and total scores show a normal distribution.

### Confounding variables

We first excluded multiple births from the dataset, to remove any possible familial clustering effects, and controlled for gender, SES, parity and birthweight. Gender is a major risk factor for depression in adolescent girls, [21] and birthweight was used as a general proxy for a child’s general health, to adjust for fundamental physiological problems which could influence both the development of RAP and later psychiatric problems. Our rationale came from Apley’s original study, [2] which surveyed 1000 children, and showed that the only physical difference was RAP school children weighed 1 to 2 lbs lighter [2]. As RAP was determined from maternal reports, we controlled for maternal education and pre-exposure maternal anxiety and somatic scores, both measured during pregnancy (32 weeks gestation). We also controlled for maternal depression (measured 8 weeks after birth) as previous research has shown all of these measures to be highly correlated over time.[18] [15]

Finally, we controlled for pre-existing child psychopathology. Mothers reported on children’s behavioural and emotional problems at 9 and 11 years with the Strengths and Difficulties Questionnaire (SDQ), a widely used and validated screening questionnaire that was developed from the Rutter questionnaires. [22] [23] The SDQ consists of 25 questions that are divided into 5 subscales (emotional problems, hyperactivity, conduct problems, peer problems, and prosocial score). The first 4 subscale scores can be combined to give a total difficulties score. For a more clinically meaningful comparison, we analysed the 2 groups by looking at the highest scorers on each scale, to see whether children with RAP were more or less likely to be those most comparable to a clinically disturbed group. For both scales, the top 10% of values were included, because this represents the recommended use of the SDQ. High scores on the SDQ have been shown to be predictive of psychiatric disorders among children (specificity: 94.6%; sensitivity: 63.3%). [23]

### Statistical analysis

Logistic regression models were used to examine associations between RAP at 4 different time points, and self-reported depression or anxiety symptoms on the CIS-R at 18 years. We dichotomised both RAP (to create a binary indicator of 5 or more episodes of pain in the past year) and CIS-R score (score above/ equal to 12 indicates depression and/or anxiety disorder). All analyses were conducted using Stata version 13.

For the primary analysis, our exposure variable was the total RAP score, combining scores from all 4 different time points. We then analysed separate models, to explore the association between each of the different RAP scores in turn (at age 3, 4, 7 and 9 years) and depression and anxiety at age 18. Initially, unadjusted associations were examined, and then adjusted for potential confounders.

To investigate the moderation effects of cognitive style, an interaction term between RAP and cognitive style was included. Analysis was conducted to compare models with and without interaction terms, testing moderation effects of cognitive style using likelihood ratio tests. In order to maximise power to look at effect modification, we took a median split and created two equal groups, those with the highest 50% of scores on the Cognitive Style Questionnaire (CSQ) and the 50% with the lowest scores on the CSQ. We did not use a clinically defined cut-off of cognitive style. Thus, we used a binary high vs low variable, and looked at a relatively higher cognitive style, within the normal range.

Due to missing data on exposure, outcome and confounding variables, the samples used for the complete case analysis were substantially reduced in size compared with the original starting sample. Thus, if we restricted our analyses to only the complete case data, it could lead to biased or underestimated results. To overcome this problem, we examined the potential impact of missing data on our findings by conducting a sensitivity analysis on our primary outcome using multiple imputation by chained equations. [24]There were 6,144 children with complete data across all the exposure measures and this was used in the complete case analysis. However, there were a total of 8368 children who had partial data but missed some variables. For these children (8368–6144) we used partial data to impute missing data.

## Results

32% of mothers reported their child has having 5 or more episodes of abdominal pain in the past year, on at least one occasion (1,978/ 6,144). 15.3% of the cohort, who remained in the study, met criteria for depression and/or any anxiety disorder on the CIS-R at 18 years. Table 1 provides descriptive information about the participants with complete case and partial data for the exposure and outcome variables.

**Table 1:** Sample characteristics for participants with complete case and partial data.

Variable	Total RAP by age 9 (N = 6,144)	With Total RAP & CISR (N = 3,003)	With Complete Case & CISR (N = 2,474)
RAP at 3 yrs (N, %))	384 (3.86%)	113 (3.76%)	88 (3.56%)
RAP at 4 yrs (N, %)	614 (6.95%)	238 (7.93%)	189 (7.64%)
RAP at 7 yrs (N, %)	1,237 (17.32%)	561 (18.68%)	471 (19.04%)
RAP at 9 yrs (N, %)	738 (12.01%)	392 (13.05%)	328 (13.26%)
CISR depression/anxiety at 18 yrs (N, %)	434/3,003 (14.5%)	434/3,003 (14.45%)	336/2474 (13.58%)
Social Class (N, % in 1)	400/6,065 (6.60%)	247/2,990 (8.26%)	235 (9.50%)
Mat Education (N, % A’Level/ Above)	2,618/6,065 (43.17%)	1,491/2,990 (49.87%)	1,287 (52.02%)
Maternal Anxiety (N, % high scorers)	1,176/6,065 (19.14%)	511/ 3,003 (17.02%)	406 (16.41%)
Maternal EPDS depression at 8wks (N, %)	819/6144 (13.33%)	350/3,003 (11.66%)	241 (9.74%)
Maternal Somatic Score (N, % high scorers)	1,061/6,144 (17.27%)	516/3,003 (17.18%)	409 (16.53%)

### Recurrent abdominal pain & depression and anxiety at age 18

Children with RAP were at increased risk of developing depression and anxiety disorders at age 18, and this remained after adjusting for confounding variables. Table 2 illustrates the effect of various adjustment models on the odds ratios for RAP at 3, 4, 7 & 9 years as a predictor of emotional distress disorders at age 18. Each additional time-point of RAP was associated with a 26% (CI: 7% to 47%) increase in odds of being diagnosed with depression or anxiety at 18. The findings were unchanged when we conducted the analyses using the imputed datasets. Moreover, RAP acts as an independent and specific risk factor for adverse outcomes in adulthood, over and above existing psychopathology. In this study, the association between RAP and adverse psychological outcomes in adulthood remains, even after adjustment for emotional and behavioural problems at ages 9 and 11 years. (OR: 1.23; 95% CI: 1.03–1.47).

**Table 2:** Odds ratios for recurrent abdominal pain (RAP) at 3, 4, 7 & 9 years as a predictor of depression &/ anxiety at 18.

Recurrent Abdominal Pain (RAP) at different ages	OR (95% CI), p value (Crude) (N = 3003)	OR (95% CI), p value for Complete Case sample (N = 2474)	OR (95% CI), p value with adjustments (1)	OR (95% CI), p value with adjustments (2)	OR (95% CI), p value with adjustments (3)	OR (95% CI): Multiple Imputation with confounders (n = 8368)
RAP at 3 years	1.63 (1.03, 2.06), p = 0.038	1.32 (0.75, 2.33), p = 0.336	1.10 (0.62, 1.95), p = 0.738	1.11 (0.63, 1.98), p = 0.715	1.05 (0.59, 1.88), p = 0.850	1.50 (1.04–2.15), p = 0.03
RAP at 4 years	1.60 (1.15, 2.23), p = 0.005	1.62 (1.11, 2.36), p = 0.0.013	1.58 (1.08, 2.32), p = 0.020	1.59 (1.08, 2.34), p = 0.017	1.53 (1.04, 2.25), p = 0.03	1.36 (0.97–1.90), p = 0.07
RAP at 7 years	1.22 (0.95, 1.58), p = 0.113	1.29 (0.98, 1.70), p = 0.073	1.25 (0.94, 1.66), p = 0.118	1.25 (0.94, 1.65), p = 0.123	1.19 (0.90, 1.59), p = 0.210	1.14 (0.93–1.40), p = 0.22
RAP at 9 years	1.67 (1.27, 2.18), p = <0.001	1.67 (1.24, 2.26), p = 0.001	1.51 (1.11, 2.05), p = 0.008	1.53 (1.13, 2.08), p = 0.006	1.43 (1.05, 1.96), p = 0.022	1.50 (1.17–1.91), p = 0.001
Total RAP (ordinal data)	1.36 (1.12, 1.56), p = <0.001	1.37 (1.17, 1.60), p = <0.001	1.30 (1.11, 1.52), p = 0.001	1.30 (1.11, 1.53), p = 0.001	1.26 (1.07, 1.47), p = 0.005	1.25 (1.09–1.36) p = <0.001

^(1)^ Model 1: Adjusted for gender, parity, birthweight;

^(2)^ Model 2: Adjusted for Model 1 variables, and SES & maternal education;

^(3)^ Model 3: Adjusted for Model 2 variables, as well as maternal anxiety & maternal somatic scores (derived from CCEI, Crown Crisp Experiential Index, in pregnancy) & maternal depression scores (derived from EPDS, post-natal depression scale, at 8 weeks gestation)

Our findings showed that experiencing recurrent abdominal pain (RAP) at just one age significantly increased the risk for emotional distress disorders during young adulthood (OR = 1.41 (1.09–1.83) p = 0.01). Moreover, as chronicity of RAP stretched across different ages, the risk for later emotional distress disorders further increased. The dose-response relationship is illustrated in Table 3 which shows the percentage (and odds ratios) of participants who go on to become depressed/ anxious with each increased episode of RAP. Although the confidence intervals widen as the number of participants in each group decreases, as there is strong evidence for a linear trend.

**Table 3:** The percentage (and odds ratios) of participants who go on to become clinically depressed/ anxious with each increased time point of RAP.

	No RAP	RAP x 1 time point	RAP x 2 time points	RAP x 3 or more time points
**Percentage (%)**	12.5%	17.6%	18.5%	29.3%
**Odds Ratio (OR)**		1.41 (1.09–1.83) P = 0.01	1.42 (0.92–2.19) P = 0.12	2.28 (0.93–5.62) P = 0.07

### Impacts of cognitive style

Our analysis demonstrated that a negative cognitive style moderates the association between recurrent abdominal pain in childhood and later emotional distress disorders by amplifying the risk. There was statistical evidence for an interaction term, where the model with an interaction better fits the observed data (LR Chi2 = 11.34; p = 0.0008) (see Table 4) and in the stratified analysis there was evidence for an association between RAP and depression and anxiety if the child has a high negative cognitive style.

**Table 4:** Relationship between negative cognitive style and the risk of a person with RAP developing depression/ anxiety.

Exposure	Entire Sample OR (CI), p value	High Negativity OR (CI), p value	Low Negativity OR (CI), p value	Test for Interaction—LR Chi Squared Test
**Total Number of RAP**	1.36 (1.12–1.56) P = 0.000	1.55 (1.25–1.92) P = 0.000	0.72 (0.45–1.05) P = 0.09	LR Chi2 = 11.34 P = 0.0008

**Fig 2:**
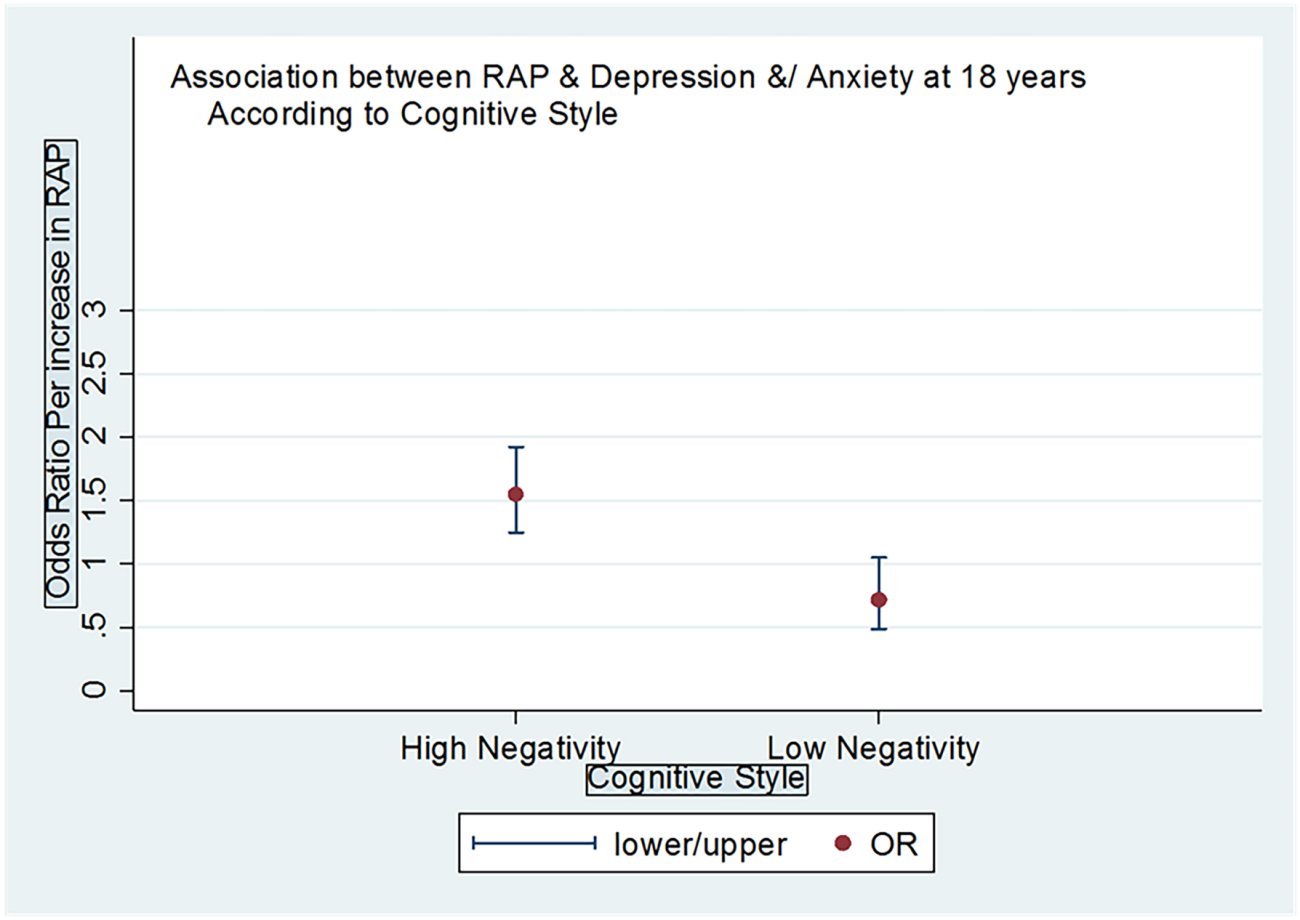
Graph depicting that a negative cognitive style amplifies the potential for emotional distress disorders in early adulthood after childhood RAP.

Table 5 and Fig 2 further illustrate how a negative cognitive style amplifies the relative risk of a person with childhood RAP developing an emotional distress disorder at 18 years. Of those with no RAP, 17.3% of those with a negative cognitive style go on to develop depression/ and anxiety in adulthood, whereas when they have both RAP and a negative cognitive style, 26.4% (i.e. over a quarter of them) will develop later emotional disorders.

**Table 5:** The cognitive style groups (high versus low) and the *relative risk* of depression/ anxiety in those with RAP.

	High CSQ	Low CSQ
**No RAP**	17.30% (142/821)	7.5% (66/877)
**RAP (at least once)**	26.4% (120/454)	7.86% (33/280)

## Discussions

In this analysis of the ALSPAC cohort, we show that individuals who suffer from recurrent abdominal pain (RAP) in childhood have an increased risk of developing depression and/or anxiety disorders at 18. We found a dose-response relationship between chronicity of RAP and risk of later emotional distress disorders, and this relationship persisted even after adjusting for a range of potential confounders, including existing psychopathology. By analysing a large database with detailed information on both exposure and outcome measures, our findings replicate previous longitudinal studies on smaller samples [6, 15, 25–27] and confirm the role of childhood RAP as an independent and specific risk factor, above and beyond pre-existing psychopathology, for emotional problems in later life.

Furthermore, this is the first study to explore how a negative cognitive style potentially moderates the association between recurrent abdominal pain in childhood and later emotional distress disorders, by amplifying the risk. The currently accepted biopsychosocial model of chronic pain states that biological, psychological and social factors interact with one another to influence the subjective experience of pain.(29) This approach is essential to the clinical understanding of the complex interplay between dysfunction, pain, disability and distress.(30) Whether or not cognitions need to be pain-specific to impact the pain experience is currently unknown. (12) Our study suggests that even a general cognitive style (as measured by the CSQ) could be important.

### Implications for practice

Recurrent abdominal pain causes considerable distress to children and their parents and it is an important public health concern with notable cost implications.(29) Moreover, emotional distress disorders in young adulthood can set the tone for a lifetime of interpersonal, functional and economic difficulties. Thus, mitigating depression and anxiety during this important developmental period should be a priority. Our findings suggest that clinicians might view childhood RAP as a potentially useful clinical risk marker to predict later emotional problems. It is easy to detect and carries less stigma compared to other markers of risk, including parental psychopathology and maltreatment.(27).

Our finding that a highly negative cognitive style amplifies the potential for emotional distress disorders in early adulthood after childhood RAP, has implications for clinical practice. Since our findings are cross sectional (cognitive style was measured at 18 years) we cannot draw any causal conclusions from this data, but epidemiological studies indicate that cognitive styles are trait like and therefore likely to be stable from early life.(14) (21) Earlier measures of cognitive style would have assisted us in understanding its moderating potential, but our results highlight an important role for cognitive factors in both paediatric chronic pain and internalizing disorders. Our findings suggest that even a general cognitive style may be important understanding the pain experience. Moreover, our findings could offer a framework to help us understand pain-specific cognitions, and how they might be generalised to other areas of our lives. For example, a doctor could decipher a child’s cognitive misappraisals about their abdominal pain, by categorising them into how they feel about themselves, the world and their future. If they demonstrate a highly negative cognitive style, they could be prioritised for psychological treatment.

Medical practitioners should offer focused investigations to rule out explanatory pathology, and remain aware that biological, psychological and social factors can all impact on the development and recognition of abdominal pain.(30) The mind-gut connection is a rapidly expanding field and studies on patients with functional abdominal pain indicate that emotional stress can cause molecular changes, including the upregulation of pain-generating pathways.(31) The strongest evidence for effective therapy comes from psychological treatments, which aim to reduce psychological stressors driving the brain–gut axis.(30) Cognitive Behavioural Therapy (CBT) can successfully treat functional conditions in both children and adults (32) and trial studies show that CBT which directly targets visceral sensations are particularly effective.(33) Helping children and parents understand that functional symptoms are due to potential visceral hypersensitivity, which can be modified, and not manifestations of disease, is the cornerstone of treatment. These interventions must be given in conjunction with family support, as there is substantial evidence that parental psychological responses to a child’s pain (e.g. catastrophizing) strongly influence a child’s attention and interpretation of pain.[28–30] Thus, a carefully targeted, systemic CBT intervention to help children mitigate their abdominal pain could offer them a tangible framework upon which to build emotional resilience and thus, alter their risk trajectory.

### Strengths and weaknesses of this study

These findings need to be interpreted in light of some limitations. As with any large population based longitudinal study, sample attrition is a problem, which in this cohort is strongly associated with socioeconomic disadvantage. We used multiple imputation techniques to account for missing data, but this technique is still limited by our pre-existing knowledge of which factors to explore.

Information bias is another potential weakness as RAP was determined by maternal report. A previous ALSPAC study showed that mothers with high anxiety are more likely to report RAP in their children, but they are also less likely to return the questionnaires, making the relationship between maternal anxiety and RAP less demonstrable.[15] However, when we adjusted our analyses for pre-existing maternal anxiety, this did not substantially alter the results.

We adjusted for a wide range of possible influential factors, but as with any observational study, residual confounding cannot be ruled out. By adjusting for maternal factors, we accounted for some of the genetic contribution, but the role of shared genetic vulnerability remains a plausible mechanism to explain some of the association and can be tested in future studies. Also, we did not explore a biological basis to RAP but previous research strongly indicates that very few cases are caused by organic disease.[15] [25] By adjusting for birth weight, guided by Apley’s original study, we controlled for some of the more relevant factors.[25]

Finally, the ALSPAC sample is a predominantly white British cohort, but previous studies have found differences in the prevalence of somatic symptoms between white and non-white ethnic groups. [31] It is thought that some cultures are more reserved in expressing feelings of distress, and requests for medical attention for bodily symptoms being more imperative than purely psychological ones. Future research to compare childhood RAP between international cohorts could be valuable.

To our knowledge, this is the first study to provide evidence for an influence of cognitive style on the relationship between childhood recurrent abdominal pain and emotional distress disorders in adulthood. The strengths of our study are the large sample size, prospective collection of data on RAP and confounders, and the duration of follow up. Moreover, the adult measures of psychiatric diagnosis were collected blind to RAP status so there was no risk of recall bias. Furthermore, the data was collected from four time points, which allowed us to generate ordinal data in which to analyse a dose-response relationship.

## Conclusions

Recurrent abdominal pain in children is a clinically useful risk marker for predicting depression and anxiety in early adulthood, and a possible target for intervention. Our results indicate a dose-response trend between the chronicity of abdominal pain and the risk of subsequent emotional distress disorders, supporting the hypothesis of a possible causal pathway. We have also found that a negative cognitive style, in the context of recurrent abdominal pain, may amplify the risk of later emotional distress disorders. By categorising pain-specific cognitions into how a child feels about themselves, the world and their future, further research may be able to specify therapy targets (e.g. negative automatic thoughts that enter a child’s mind when he feels an abdominal sensation). Future studies are also needed to identify empirically validated maintaining factors (e.g. avoidant behaviours in the child, unhelpful social modelling from the parents, and ambiguity from healthcare professionals). To develop a theoretical account of why the harmful cognitions do not self-correct, all these factors need to be taken into consideration. This in turn will lead to the development of specialised cognitive behavioural treatments to reverse the maintaining factors and alter long term problems with emotional regulation and pain. By detecting vulnerable individuals early in life, we may be able to alter harmful illness trajectories in both pain and psychiatric domains.
